# Acute Coronary Syndrome Mimicking Takotsubo Cardiomyopathy or Takotsubo Cardiomyopathy Mimicking Acute Coronary Syndrome?

**DOI:** 10.1155/2020/6562316

**Published:** 2020-02-24

**Authors:** Martin Chaumont, Marc Blaimont, Rachid Briki, Philippe Unger, Nadia Debbas

**Affiliations:** ^1^Cardiology Department, CHU Saint-Pierre, 322 rue Haute, B-1000 Brussels, Belgium; ^2^Cardiology Department, Hôpital de Jolimont, 7100 La Louvière, Belgium

## Abstract

A healthy 66-year-old female presented to the emergency department with acute chest pain, T-wave inversion in the anterior leads, and elevated troponin-I. Coronary angiography showed a stenosis in the midportion of the left anterior descending coronary artery (LAD), which did not wrap the left ventricle (LV) apex. LV angiography demonstrated a large LV apical akinetic systolic ballooning with a 45% ejection fraction. Fractional flow reserve (FFR) of LAD lesion was 0.71. Percutaneous intervention was performed. At six months, transthoracic echocardiography was normal. Fifteen months later, the patient presented with chest pain and a small rise in troponin-I. Coronary angiogram was unchanged. Repeat FFR in distal LAD was 0.86 and left ventriculography was normal. Diagnostic criteria for Takotsubo cardiomyopathy (TTC) require the absence of obstructive coronary artery disease. In the present case, TTC was highly suspected on the basis of typical LV apex ballooning. However, significant ischemia in the same territory was demonstrated by positive FFR, which could not be falsely positive in this context. Current TTC diagnostic criteria increase specificity for diagnosing TTC. This case reminds us that it is at the price of reduced sensitivity, since there is no reason to believe that coronary lesions may protect from TTC.

## 1. Introduction

We herein present a typical case of Takotsubo cardiomyopathy (TTC) with an atypical feature: a positive fractional flow reserve in the hypokinetic territory.

## 2. Presentation of the Case

In March 2014, a 66-year-old exsmoker female presented to the emergency department with acute chest pain, shortness of breath for three hours and anterior non-ST-segment elevation myocardial infarction (NSTEMI), T-wave inversion > 3 mm in the anterior leads without significant ST shifts, and a major increase of troponin-I (150 ng/ml; normal < 14 ng/ml). She reported a medical history of hypertension and hypercholesterolemia treated with angiotensin-converting-enzyme-inhibitor and statin, respectively. Physical examination on admission was unremarkable. Loading doses of clopidogrel (600 mg), acetylsalicylic acid (300 mg), and enoxaparin (60 mg) were given; then, she immediately underwent a coronary angiography via a right radial artery approach.

**Figure 1:**
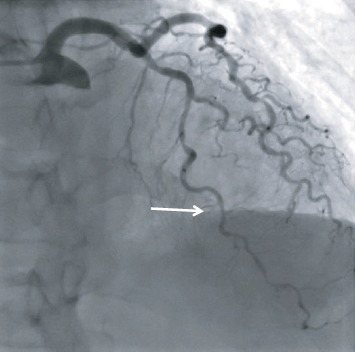
Diagnostic coronary angiogram. Intermediate lesion in the midportion of the left anterior descending artery (arrow) (right anterior oblique cranial).

**Figure 2:**
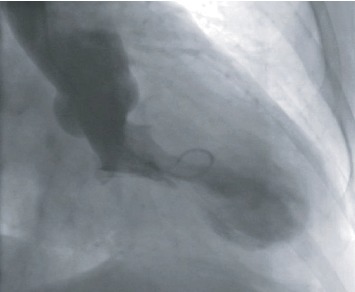
Left ventricle (right anterior oblique cranial). Large apical dyskinesia.

Coronary angiography showed a diffusely diseased and calcified left anterior descending artery (LAD) with a single intermediate lesion in its midportion as assessed by quantitative coronary angiography (Figure 1, Moving [Supplementary-material supplementary-material-1]). The left circumflex artery (LCx) and the dominant right coronary arteries were also calcified but without significant lesion (<20% at angiography). Left ventriculography demonstrated a large akinesia of apical and midportions of the left ventricle (LV) inducing apex ballooning in the systole (Figure 2, Moving [Supplementary-material supplementary-material-1]) with an ejection fraction of 45%.

**Figure 3:**
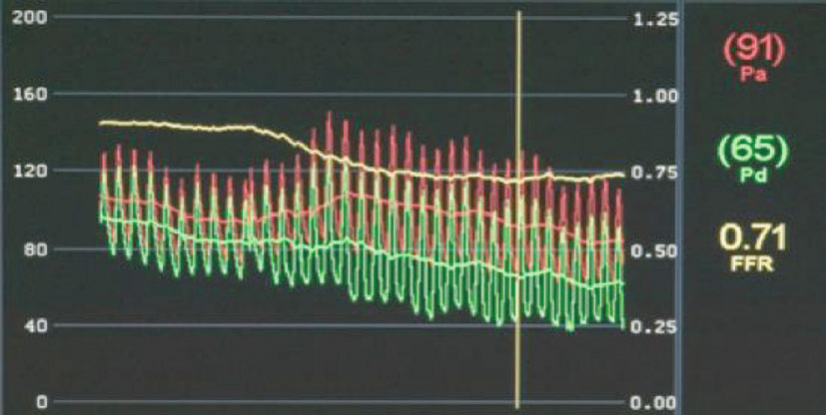
Fractional flow reserve in the midleft anterior descending artery.

In order to differentiate between a TTC and a NSTEMI resulting from the culprit mid-LAD lesion, the ischemic significance of the latter was tested with fractional flow reserve (FFR) assessment. A 6Fr FL4 guiding catheter (Boston Scientific, USA) was positioned in the left main ostium, intracoronary nitroglycerine (200 *μ*g) was injected, and PressureWire™ Certus (St. Jude Medical, USA) was advanced distally to the LAD lesion. At rest, Pd (mean blood pressure distal to the stenosis) over Pa (mean aortic pressure) was 0.83. Steady state hyperaemia was induced by continuous intravenous administration of adenosine through a 4Fr sheath placed in the humeral vein. The infusion was kept at 140 *μ*g/kg/min for 180 seconds. The patient complained of “typical” adenosine-induced symptoms, and the Pd curve displayed the U shape. FFR proved to be positive for ischemia at 0.71 in the distal LAD, and pull back revealed a pressure jump proximal to the mid-LAD lesion (Figure 3).

Should we consider this presentation to be an acute coronary syndrome (ACS) related to the mid-LAD unstable plaque being the culprit lesion and implement percutaneous intervention (PCI), which in turn carries some risk, particularly in complex cases [1, 2]? Or a TTC with a typical apical ballooning [3, 4]—which pathogenic mechanism is believed to be linked to microvascular dysfunction [5] and catecholamine cardiotoxicity [6]—with a significant but innocent concomitant mid-LAD coronary artery disease and therefore treat medically?

## 3. Actual Treatment and Management of the Case

PCI was elected; a Maverick® balloon catheter (2.0 × 15 mm, Boston Scientific, USA) was advanced to the mid-LAD lesion over the FFR wire. This proved difficult, warranting deep throat catheter engagement in the left main and proximal LAD. Several long balloon inflations achieved a very good “stent-like” result at the PCI site (Moving [Supplementary-material supplementary-material-1]). However, a retrograde dissection of the proximal LAD occurred into the left main trunk, the aortic cusp, and the proximal LCx (Moving [Supplementary-material supplementary-material-1]).

At this time, the patient reported chest pain and ST elevation in the anterior leads but was haemodynamically and rhythmically stable. The LCx was immediately wired with a Luge™ (Boston Scientific, USA). We proceeded to direct stenting of the proximal LAD, covering the retrograde dissection entry, into the left main to its ostium with a 3.5 × 28 mm drug-eluting stent (Synergy™, Boston Scientific, USA). After a proximal optimisation technique (4.5 × 8 mm Maverick® balloon catheter, Boston Scientific, USA) in the left main trunk and a final kissing into LAD and LCx arteries, complete disappearance of all contrast extravasation was obtained. Decision was made to leave LCx unstented as it had a good TIMI3 flow (Moving [Supplementary-material supplementary-material-1]). The patient recovered rapidly and was discharged three days later on full medical therapy.

Fifteen months later, the patient presented in the setting of a hypertensive crisis with chest pain, a small rise in troponin-I, and no ECG changes. Coronary angiogram was unchanged, with diffusely diseased coronary arteries, complete healing of the left main, proximal LAD, and LCx arteries, without in-stent restenosis (Moving [Supplementary-material supplementary-material-1]). There was no mid-LAD focal lesion, and the FFR in distal LAD was negative for ischemia (0.86). Left ventriculography was normal (Moving [Supplementary-material supplementary-material-1]).

## 4. Discussion

TTC and ACS involving the LAD can both induce reversible LV apical akinesia/dyskinesia [3, 4] reflecting myocardial stunning [7] and affect patients in the same age group and with similar risk factors [6].

Unlike in ACS, there is usually no significant coronary artery obstruction or acute plaque rupture [6] in TTC. However, true TTC may occur in the presence of significant CAD or even ACS, but this combination, when affecting LV apex, is particularly tricky [6, 8]. The current NSTEMI presentation supported by the positive FFR at the level of the mid-LAD partial occlusion can induce transient apical LV stunning and ballooning through several mechanisms, including coronary microvascular dysfunction which is encountered similarly in TTC and in ACS [5].

The age and gender, the postmenopausal context, and the transient large apical and midventricular segments wall motion dysfunctions, thus extending beyond a single epicardial coronary distribution may favor the diagnosis of TTC [6]. However, the lack of history of stressful event, the normal corrected QT interval, the high troponin-I level [6], and the long LAD anatomy encompassing anterior-apical and inferior-apical territories support the NSTEMI diagnosis. Moreover, according to the current cardiology society criteria, the positive FFR value in the mid-LAD allows to rule out the latter diagnosis [6]. Blunted coronary vasodilation in ACS and in TTC may decrease the sensitivity but certainly not the specificity of the FFR [2]. Moreover, the excellent angiographic result of sole balloon angioplasty of the culprit lesion supports the ACS diagnosis [1]. Of note, FFR measurement is safe in patients following NSTEMI [9].

When complications occurred, haemodynamic stability as well as proximal location of the dissection in a large vessel prompted us to proceed with interventional treatment rather than surgery despite the involvement of left main and aortic cusp. The mid-LAD culprit lesion was left unstented—with an angiographic “stent-like” result and complete revascularisation—because of the potential difficulty foreseen in advancing a stent to cover it. Complete healing 15 months later further confirms the treatment adequacy.

## 5. Conclusion

This case emphasizes an uncommon diagnostic challenge the clinician may be facing. In this setting, FFR was crucial to assess the functional significance and the aetiological role of the focal mid-LAD lesion, although a combined aetiology (ACS followed by TTC or the reverse) cannot be ruled out.

## Supplementary Materials

Supplementary Materials Moving Image 1: diagnostic coronary angiogram (right anterior oblique cranial). Moving Image 2: diagnostic left ventriculography (right anterior oblique). Moving Image 3: percutaneous intervention (right anterior oblique cranial). Moving Image 4: left anterior descending artery dissection (right anterior oblique cranial). Moving Image 5: (A) percutaneous treatment of the left anterior descending artery, left main, and aortic cusp dissection; (B) final result (right anterior oblique cranial (A) and caudal (B)). Moving Image 6: fifteen months later (right anterior oblique cranial (A) and caudal (B)). Moving Image 7: ventriculography fifteen months later (right anterior oblique cranial).
