# Supplementary material for: Acute Coronary Syndrome Mimicking Takotsubo Cardiomyopathy or Takotsubo Cardiomyopathy Mimicking Acute Coronary Syndrome?
Source: Case Rep Cardiol. 2020 Feb 24;2020:6562316. doi: 10.1155/2020/6562316 (PMC7060413; doi:10.1155/2020/6562316)
Supplement: Supplementary Materials — Moving Image 1: diagnostic coronary angiogram (right anterior oblique cranial). Moving Image 2: diagnostic left ventriculography (right anterior oblique). Moving Image 3: percutaneous intervention (right anterior oblique cranial). Moving Image 4: left anterior descending artery dissection (right anterior oblique cranial). Moving Image 5: (A) percutaneous treatment of the left anterior descending artery, left main, and aortic cusp dissection; (B) final result (right anterior oblique cranial (A) and caudal (B)). Moving Image 6: fifteen months later (right anterior oblique cranial (A) and caudal (B)). Moving Image 7: ventriculography fifteen months later (right anterior oblique cranial). [file 6562316.f1.pdf]

Supplementary data

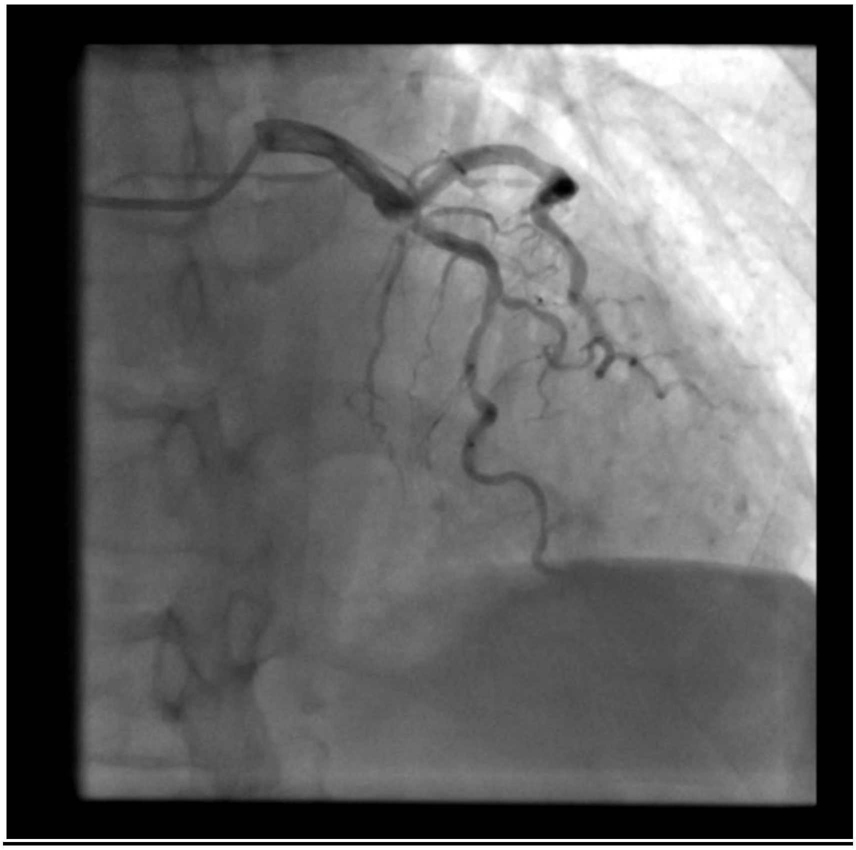

Moving Image 1. Diagnostic coronary angiogram (Right anterior oblique cranial).

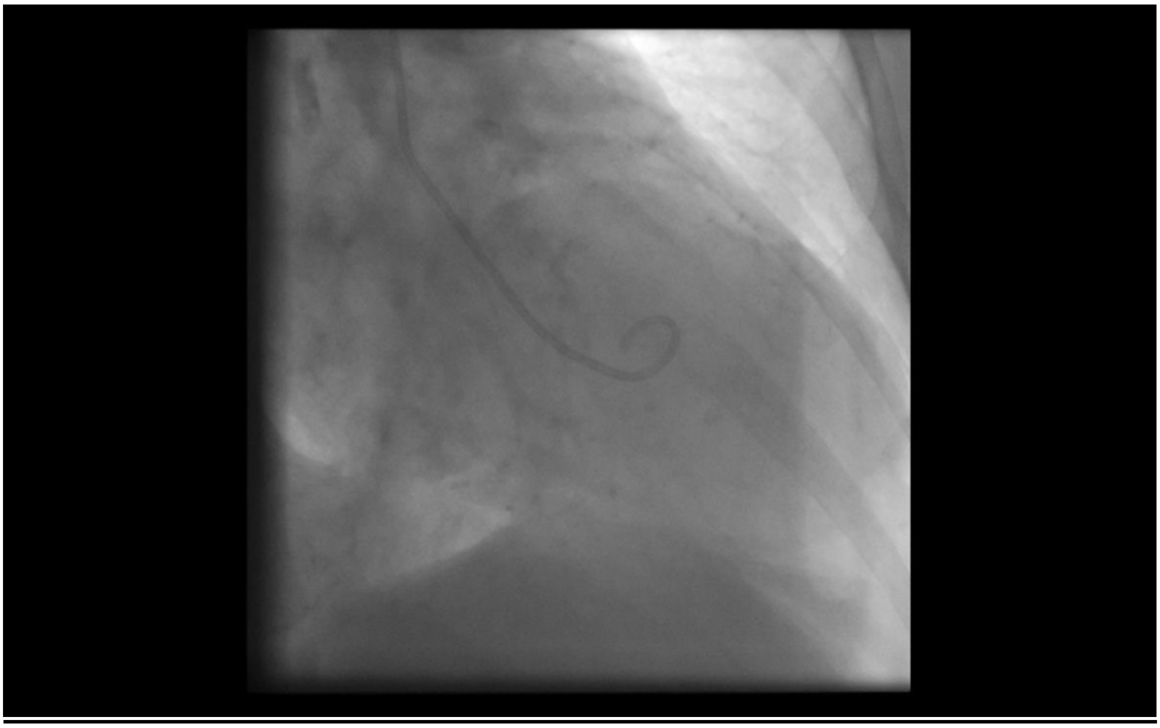

Moving image 2. Diagnostic left ventriculography (Right anterior oblique cranial).

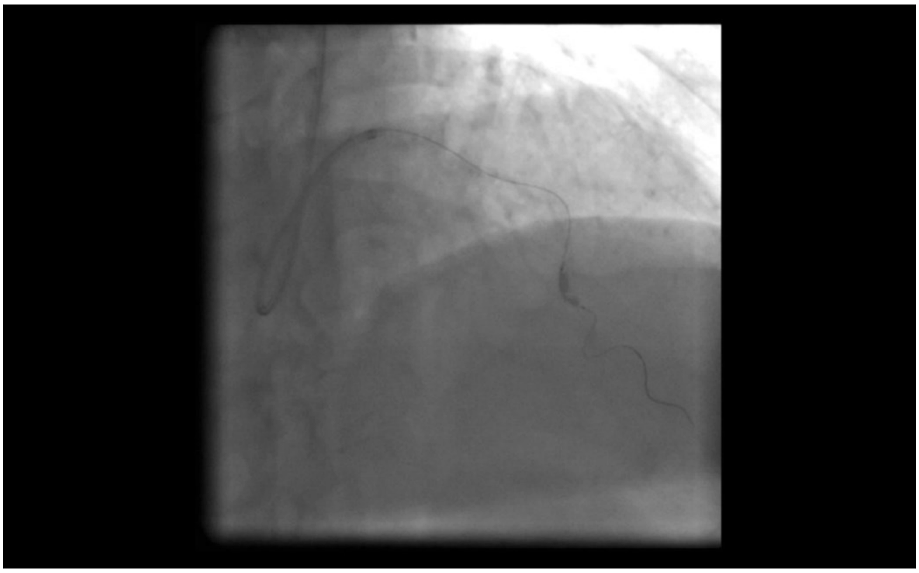

Moving image 3. Percutaneous intervention. (Right anterior oblique cranial).

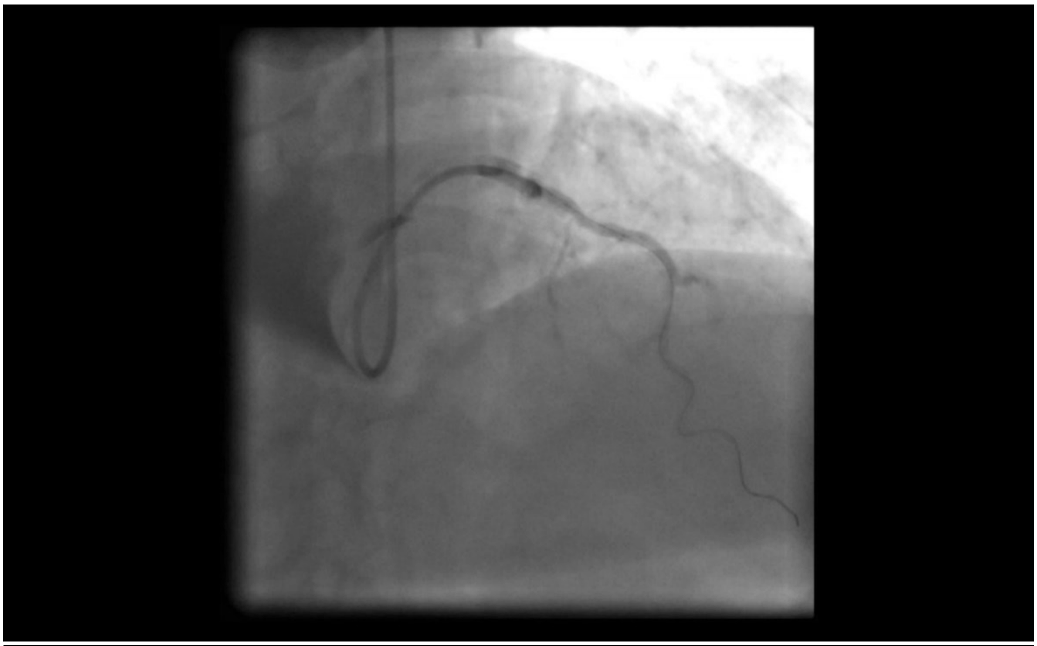

Moving image 4. Left anterior descending artery dissection (Right anterior oblique cranial).

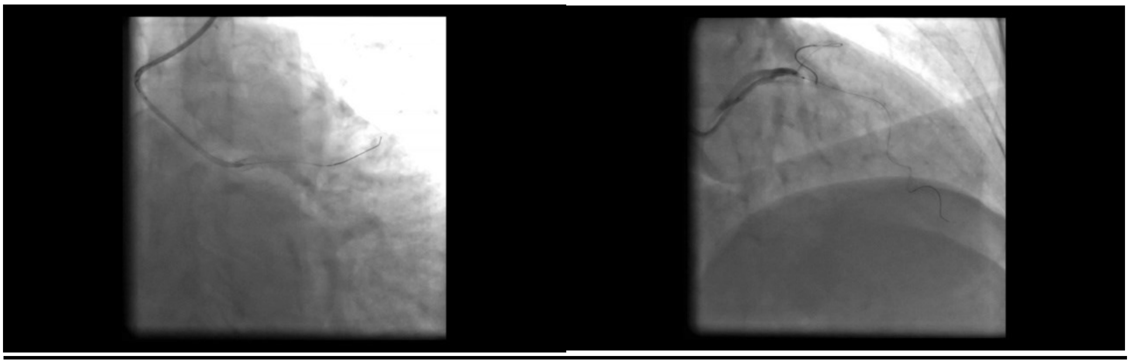

Moving images 5. A: Percutaneous treatment of the left anterior descending artery, left main and aortic cusp dissection B: Final result. (Right anterior oblique cranial (A) and caudal (B)).

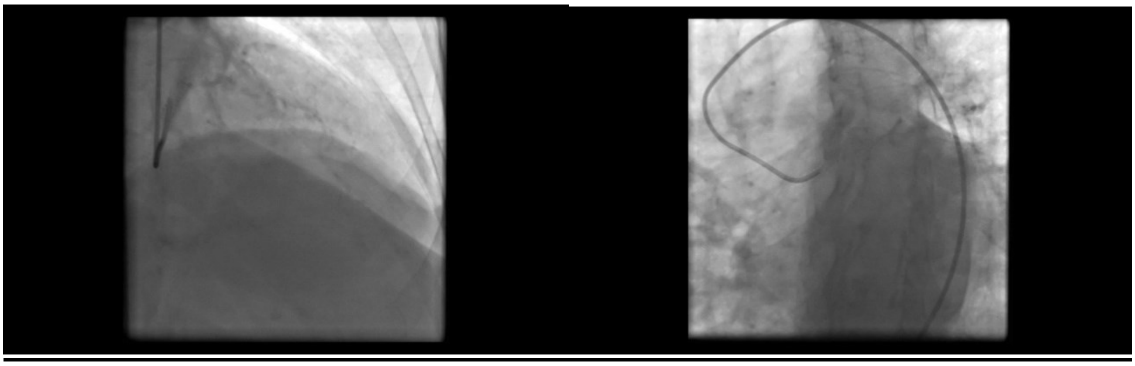

Moving image 6. Fifteen months later (Right anterior oblique cranial (A) and caudal (B)).

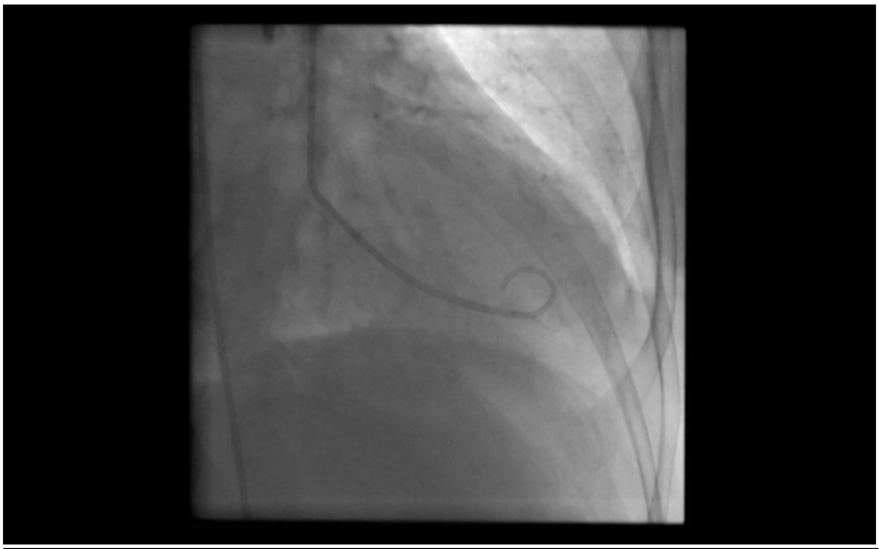

Moving image 7. Ventriculography fifteen months later (Right anterior oblique cranial).
